# Using Multi-Task Learning-Based Framework to Detect ST-Segment and J-Point Deviation From Holter

**DOI:** 10.3389/fphys.2022.912739

**Published:** 2022-06-29

**Authors:** Shuang Wu, Qing Cao, Qiaoran Chen, Qi Jin, Zizhu Liu, Lingfang Zhuang, Jingsheng Lin, Gang Lv, Ruiyan Zhang, Kang Chen

**Affiliations:** ^1^ Department of Cardiovascular Medicine, Ruijin Hospital, Shanghai Jiao Tong University School of Medicine, Shanghai, China; ^2^ Shanghai Digital Medicine Innovation Center, Ruijin Hospital, Shanghai Jiao Tong University School of Medicine, Shanghai, China; ^3^ Center for Translational Medicine, Ruijin Hospital, Shanghai Jiao Tong University School of Medicine, Shanghai, China

**Keywords:** holter, electrocardiogram, ST-Segment, deep learning, multi-task learning

## Abstract

Artificial intelligence is increasingly being used on the clinical electrocardiogram workflows. Few electrocardiograms based on artificial intelligence algorithms have focused on detecting myocardial ischemia using long-term electrocardiogram data. A main reason for this is that interference signals generated from daily activities while wearing the Holter monitor lowered the ability of artificial intelligence to detect myocardial ischemia. In this study, an automatic system combining denoising and segmentation modules was developed to detect the deviation of the ST-segment and J point. We proposed a ECG Bidirectional Transformer network that applied in both denoising and segmentation tasks. The denoising model achieved RMSE_de_, SNR_imp_, and PRD values of 0.074, 10.006, and 16.327, respectively. The segmentation model achieved precision, sensitivity (recall), and F1-score of 96.00, 93.06, and 94.51%, respectively. The system’s ability to distinguish the depression and elevation of the ST-segment and J point was also verified by cardiologists as well. From our ECG dataset, 103 patients with ST-segment depression and 10 patients with ST-segment elevation were detected with positive predictive values of 80.6 and 60% respectively. Using Holter ECG and transformer-based deep neural networks, we can detect subtle ST-segment changes in noisy ECG signals. This system has the potential to improve the efficacy of daily medicine and to provide a broader population-level screening for asymptomatic myocardial ischemia.

## 1 Introduction

Cardiovascular disease management is becoming increasingly standardized, such as by establishing chest pain centers and improving regional collaborative treatment networks. However, at least 290 million Chinese people are suffering from cardiovascular diseases, particularly ischemic heart disease (IHD), and the morbidity and mortality of cardiovascular diseases are increasing annually (Du et al., 2019; Ma et al., 2020). There are two points that cannot be ignored. The awareness rate of IHD risks is lower than the prevalence rate (Garrido et al., 2020; Daponte-Codina et al., 2022), and the difficulty in treating ischemia comes from poor regeneration of cardiomyocytes after IHD and myocardial infarction (MI). Although the myocardium of the patients with chronic coronary syndrome has been damaged, the tolerance of myocardial cells to ischemia increases due to the formation of coronary collateral circulation. ST-segment changes of chronic coronary syndrome usually appear on the ECG when the patients have increased oxygen consumption of the body, such as during exercise, while ST-segment changes of acute myocardial infarction can appear when patients are at rest. However standard ECG records myocardial electrical activity when patients are in a calm state, such that an abnormal ECG is less likely to be identified. Holter has the advantage of recording heart electrical activity for longer periods, and the ischemic alterations seen on Holter simultaneously during chest pain bouts can assist in the diagnosis of angina. It also offers higher diagnostic performances for painless myocardial ischemia as well. Therefore, long-term monitoring and early detection are critical.

Artificial intelligence (AI) has presented its ability to solve complex and time-consuming problems, freeing cardiologists from their heavy lifting. Our previous research (Du et al., 2021) had proposed an FM-ECG AI-based model to identify various cardiac abnormalities in 12-lead standard ECG data. Furthermore, we believe that large-capacity long-term dynamic electrocardiograms, Holter, are better suited to AI algorithms for precisely analyzing every heartbeat to manually interpreting IHD from such a large volume of ECG data, which is a time-consuming task. Various automated algorithms for identifying IHD and MI have been advocated because of the in-depth integration of AI in medicine. Tadesse et al. (2021) proposed an end-to-end algorithm for identifying the time occurrence of MI using a 10 s 12-lead ECG. Their model could classify normal, acute, recent, and old onset cases of MI, with AUROCs of 96.7, 82.9, 68.6, and 73.8%, respectively. Cho et al. (2020) developed an algorithm to classify MI and non-MI using 12-lead and 6-limb lead ECG data (500 Hz, 10s) with AUROCs of 0.902 and 0.880, respectively. Zhao et al. (2020) developed an algorithm to detect ST-segment elevated myocardial infarction (STEMI) using 667 STEMI ECG data. In the comparison test, their model outperformed cardiologists. Martin et al. (2021) used lead II ECG data from the PTB-XL database to develop a Deep-LSTM network for detecting real-time MI. The proposed model achieved an accuracy, recall, and specificity of 77.12, 75.85, and 83.02%, respectively. Makimoto et al. (2020) developed a CNN to recognize MI using 289 ECG data from the PTB database. They then examined the abilities of the model and physicians to identify MI and non-MI. The CNN achieved a higher f1 and accuracy. In cardiovascular diseases, changes in the ST-segment on ECG are closely related to myocardial ischemia. Xiao et al. (2018) proposed a CNN model to detect ST changes for examining ischemia using ECG data selected from the long-term ST Database that contains 65 24 h two-and fifteen three-lead ambulatory records. Their CNN model achieved an AUC, sensitivity, and specificity of 89.6, 84.4, and 84.9%, respectively.

The studies mentioned above have contributed to AI-enabled ECG analysis. Some studies included coronary angiography as the gold standard for myocardial infarction (Cho et al., 2020; Zhao et al., 2020), which makes the MI training data more reliable. Moreover, we also found that most of the duration of the ECG data used for analysis was 10s. Long-term ECG can help capture discontinuous ECG abnormalities, such as the ST-segment deviation of unstable angina and other myocardial lesions. However, some challenges arise when analyzing ST-segment changes on long-term ECG. First, although detecting subtle changes in ECG waves early and with great precision is necessary to reduce the risk of acute myocardial ischemia, a significant amount of research has concentrated on arrhythmia classification rather than on MI detection (Hong et al., 2020). A main reason for this, we assume, is that interfered signals from daily activities while wearing the Holter reduced the AI’s capacity to diagnose IHD. Second, 12-lead ECG data should be used to diagnose myocardial ischemia and MI, but some researchers have only used the single-lead ECG data. Third, although public datasets have ready-labeled and less noisy ECG signal, public data are sometimes too clean to apply to the real world owing to individual differences and the diversity and complexity of diseases. Moreover, existing publicly accepted public datasets for long-term ECG have been collected from abroad. Regional differences may affect model results.

To alleviate the problems mentioned, we collected real-world Holter ECG data, and the ECG Bidirectional Transoformer network (EBTnet), which is a transformer-based structure, was proposed to precisely detect the location and deviation of the ST-segment and J point on 12-lead Holter ECG data at the beat level and provide cardiologists with more accurate information about myocardial ischemia.

To the best of our knowledge, this is the first study to examine the prospect of combining ECG signal denoising and wave segmentation in the same model structure with exceptional accuracy to determine the position and the degree of IHD.

## 2 Materials and Methods

### 2.1 Model

#### 2.1.1 Overall Workflow

**FIGURE 1:**
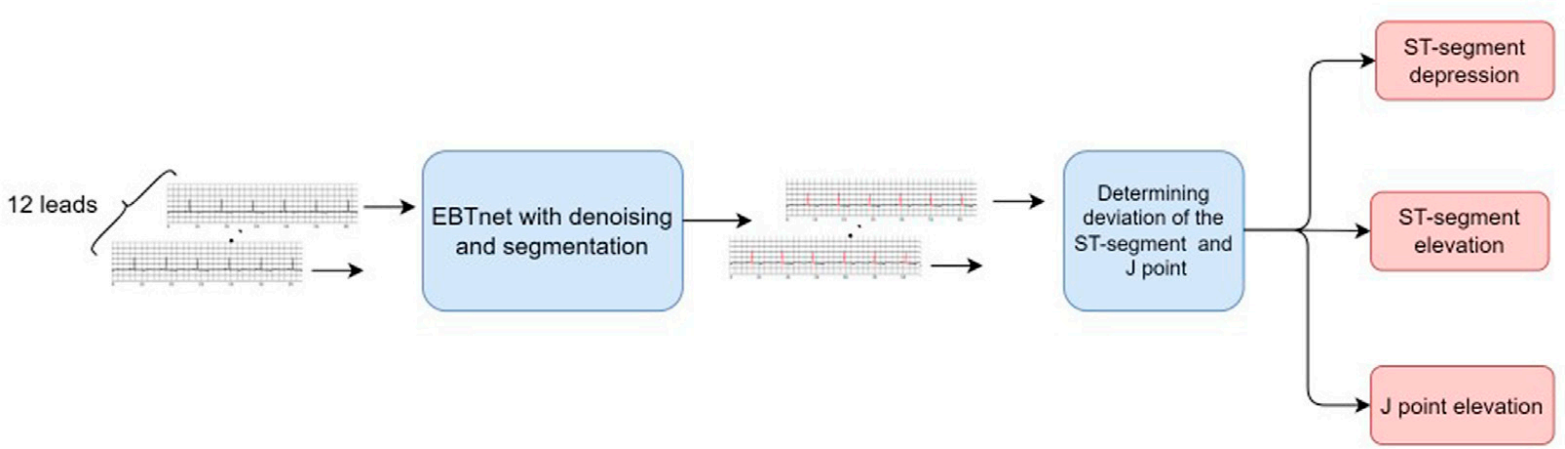
Schematic workflow of diagnosing ST-segment depression and elevation, and J point elevation from Holter electrocardiogram signal.

Figure 1 presents a schematic of the system workflow. The system starts by cropping the long-term ECG signal into patches of 7168 sampling points. In every patch, each lead is processed using the following procedures. Noises in the ECG signal is first eliminated using a denoising model, followed by a segmentation model to detect the QRS complex of the denoised ECG signal. Then, the filtered denoised QRS complex was segmented from every beat. The ST-segment and J point amplitude of deviation of each denoised QRS complex were calculated to determine any abnormal results. Abnormal results were recorded once all leads were evaluated. The pre-setting rules are used to determine the location and deviation of the ST-segment depression and elevation and J point elevation. The frequency and last times of the prediction were calculated in a straightforward manner.

#### 2.1.2 EBTnet Network Structure

In this section, we proposed EBTnet for both ECG denoising and segmentation tasks. ECG classification models usually need to capture the subtle changes in both rhythmic and waveform characteristics to improve performance. The results of ECG denoising and segmentation models are more dependent on the learning of local waveform attributes (e.g., P-waves, QRS complexes, and T waves) and less sensitive to rhythmic attributes. Inspired by a swin transformer, a shifted window attention mechanism was applied, which exhibited a strong capacity to capture feature representations in images. Our network applies one-dimensional (1D) bidireciton-shifted window-based transformer blocks (1D bidirectional SWT Blocks) to enhance the learning of ECG characteristic waveform representations.

**FIGURE 2:**
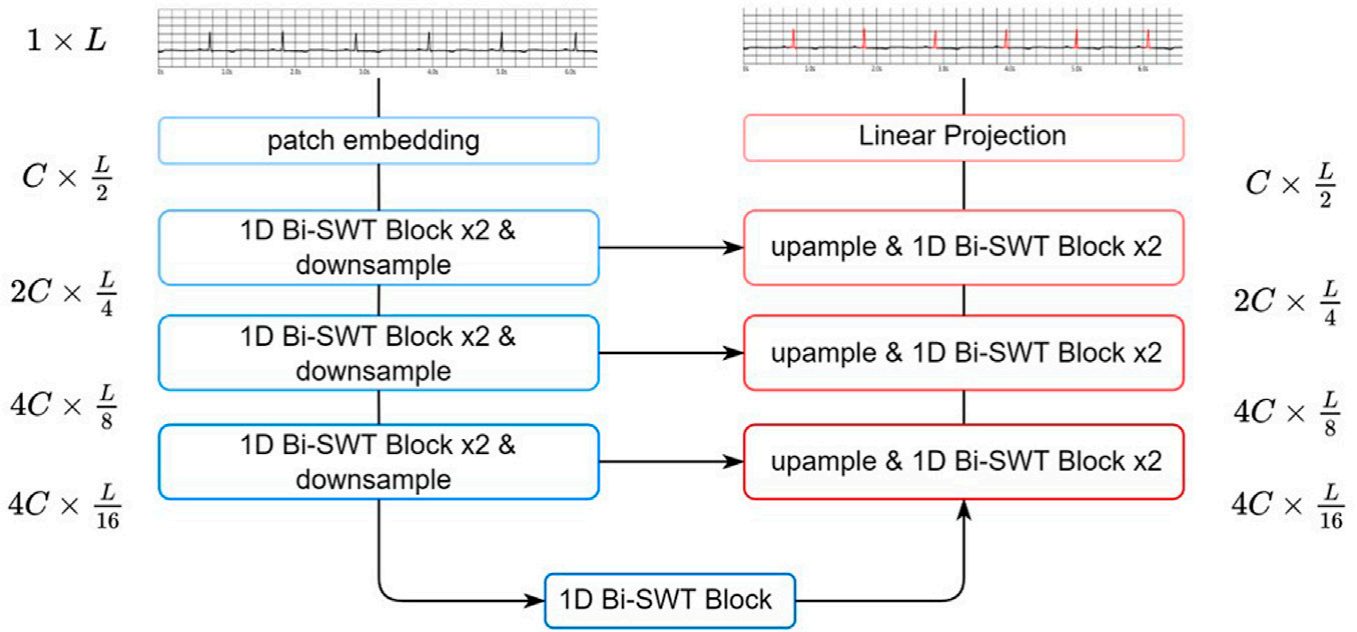
The architecture of the EBTnet.

As shown in Figure 2, EBTnet comprised an encoder, a decoder, and skip connections following a U-Net design (Ronneberger et al., 2015). Given an input ECG signal, a patch embedding layer with a stride of 2 was used to downsample the input and learn low-level features. The encoder contained a series of 1D bidirectional SWT Blocks and downsampling layers. The 1D bidirectional SWT Blocks were used to learn the relative local morphological characteristics from the ECG representational features. Downsampling layers reduced the length of ECG features, resulting in two benefits: increasing the attention field of each ECG feature patch because the window size was fixed for the entire network, and improving computational efficiency. The symmetric decoder was built with 1D bidirectional SWT Blocks, upsampling layers, and skip connections. The length of the ECG featureswas doubled by an upsampling layer, which aimed to restore the spatial information. The 1D bidirectional SWT Block in the decoder mainly fuses the upsampling features and representational features from the corresponding encoder layer through a skip connection. Eventually, the decode would restore the size of the ECG representational features from the encoder to the original input size. The last layer was a linear projection to either the denoising ECG signal task or QRS complex semantic segmentation task.

#### 2.1.3 1D Bidirectional SWT Block

A 1D SWT bidirectional block was built by extending the one-way window-partitioning strategy of the shifted window-based multi-head self-attention (SW-MSA) module from a swin transformer block using a bidirectional strategy. This shifted operation was designed to add information connections between neighboring ECG patches. However, we noticed that this connection was not fully utilized because the shift was only forward. Therefore, we added a backward shift to further increase the number of neighboring connections. The combination of the forward and backward shift directions in succession was called bidirectional.

**FIGURE 3:**
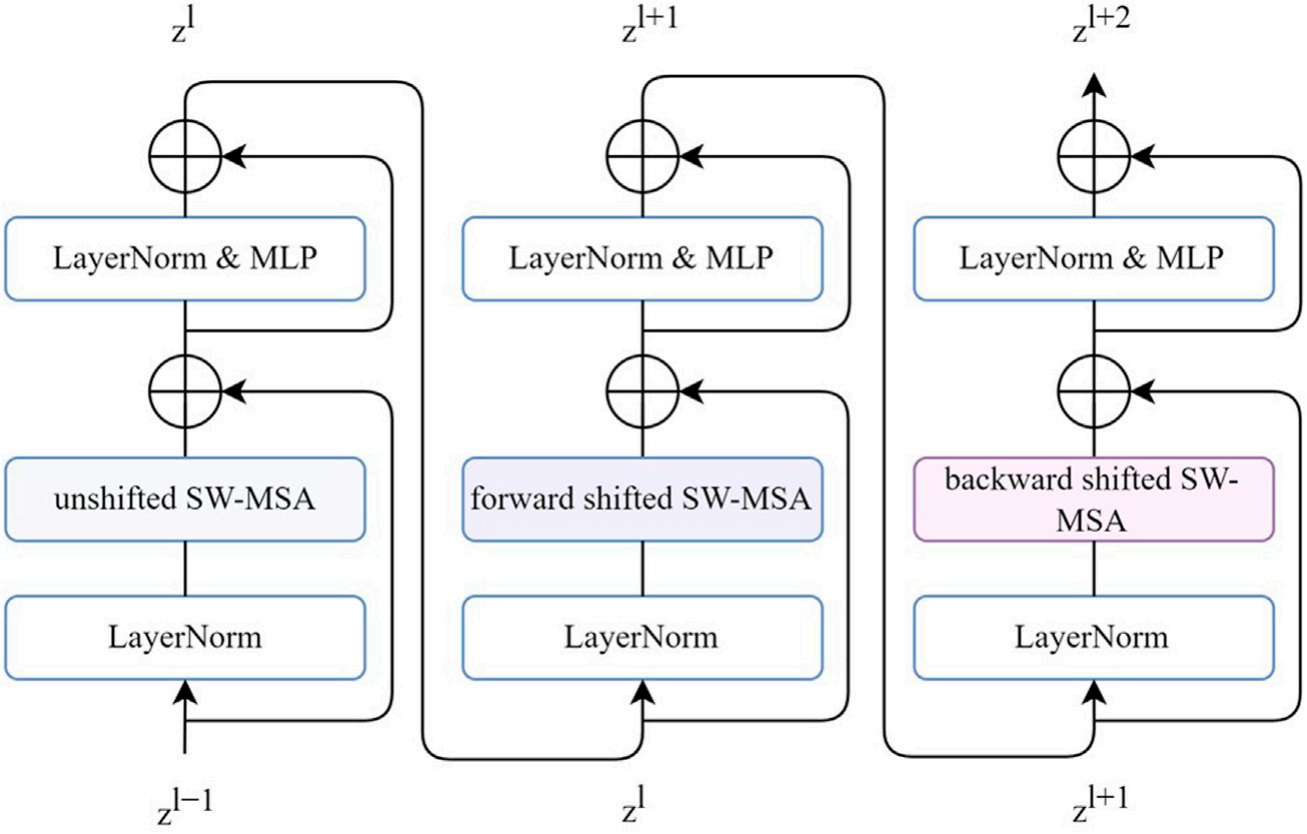
Three successive 1D bidirectional SWT blocks. Each SW-MSA is configured with unshifted, forward-shifted, backward-shifted, respectively.

Figure 3 shows three successive 1D bidirectional SWT blocks, each block built by SW-MSA, followed by two multilayer perceptron (MLP) layers with GELU non-linearity. A residual connection was applied, and LayerNorm (LN) layer was used before each MSA and MLP layer. The SW-MSA was configurated with unshifted, forward-shifted, and backward-shifted directions respectively. The transformer block can be formulated as follows:

$${\hat{z}}^{1}\;=\;W-MSA\left(LN\left({\hat{z}}^{l-1}\right)\right)\;+\;z^{l-1},$$

$$z^{l}\;=\;MLP\left(LN\left({\hat{z}}^{l}\right)\right)\;+\;{\hat{z}}^{l},$$

$${\hat{z}}^{l+1}\;=\;SW-MSA\left(LN\left(z^{l}\right)\right)\;+\;z^{l},$$

$$z^{l+1}\;=\;MLP\left(LN\left({\hat{z}}^{l+1}\right)\right)\;+\;{\hat{z}}^{l+1},$$

where ${\hat{z}}^{l}$ and $z^{l}$ are the outputs of the SW-MSA module and MLP module of block l, respectively. Self-attention was defined similarly as in previous study (Vaswani et al., 2017), which is:

$$Attention\left(Q,K,V\right)\;=\;SoftMax\left(\frac{QK^{T}}{\sqrt{d}}+B\right)V,$$

Where $Q,K,V\in \;{\mathbb{R}}^{M\times d}\;$ represent the query, key, and value matrices, respectively.

**FIGURE 4:**
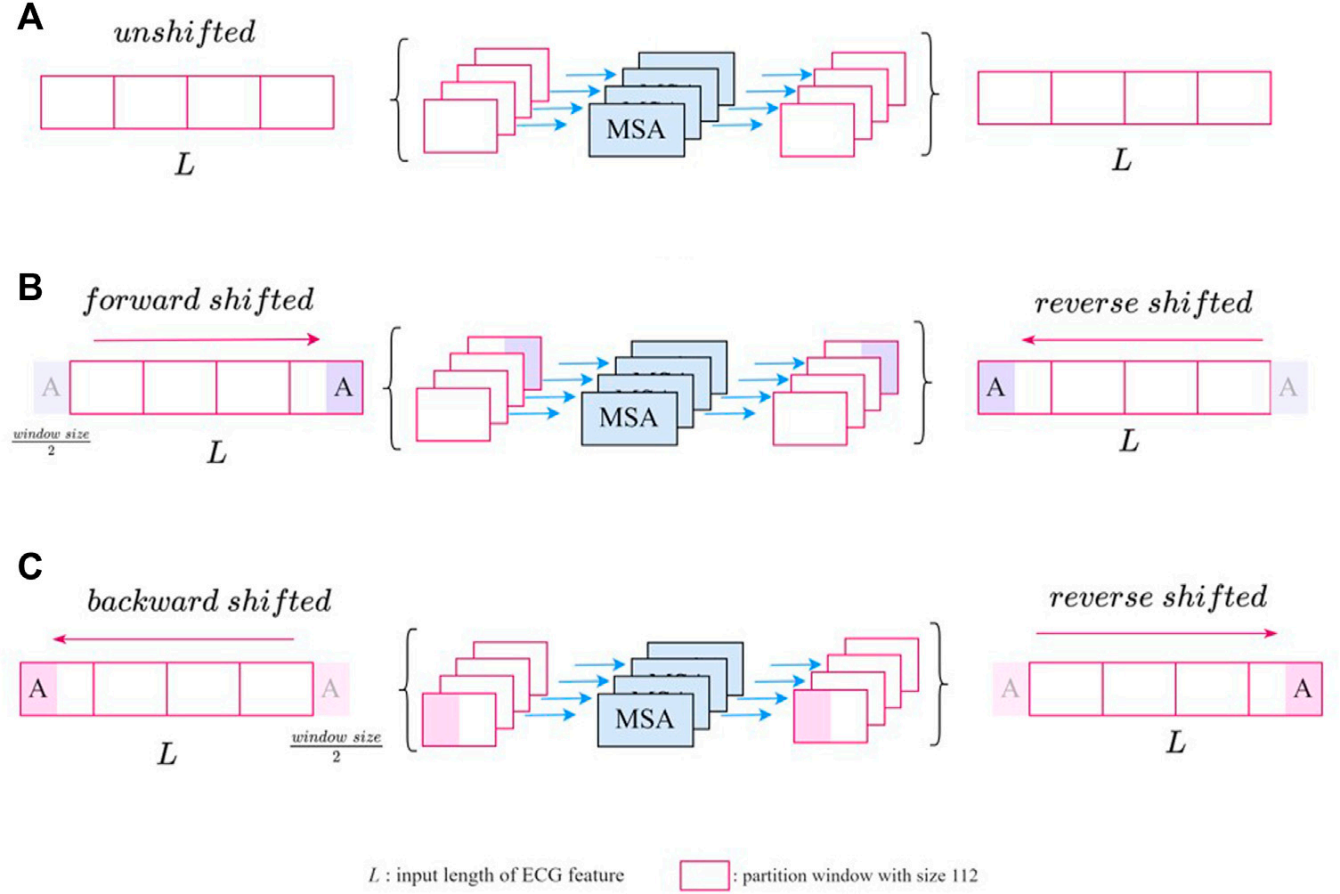
The illustration of SW-MSA module with **(A)** unshifted **(B)** forward-shifted, and **(C)** backward-shifted.

The unshifted SW-MSA is a regular window-based multihead self-attention. As shown in Figure 4A, the input feature with length L was evenly partitioned into 112 windows of size $\frac{L}{112}$ in a nonoverlapping manner. The forward-shifted SW-MSA is shown in Figure 4B, where each ECG patch was shifted forward by half of one window length, which is $56=\frac{112}{2}$ . This operation was implemented by arranging 56 lengths from the beginning to appending the ending of the feature. This was followed by regular window partitioning. Figure 4C shows the backward-shifted SW-MSA. Each ECG patch was shifted backward by half of the window. This operation is implemented by arranging 56 lengths from the end to appending the beginning of the feature. The window size parameter chosen was purely result-oriented, which details are shown in Supplementary Table S1. And the comparison between our 1D Bidirectional SWT Block and the regular SWT Block in denoising and segmentation tasks are shown in Supplementary Table S2.

#### 2.1.4 Multitask Inheritance Training Scheme

Although the denoising and segmentation tasks shared the same architecture, training was performed separately. To enhance connections between the two tasks, we applied a multitask inheritance training scheme. First, the two tasks were trained from scratch, where both the encoder and decoder use a random weight initialization. Next stage, we repeated the training task. The difference was that the weights of the encoder from each task were initialized from the weights of another task encoder in stage one. For example, the encoder weights of the model trained from the denoising task in first stage were used as the initialization encoder weights of the segmentation task model in the next stage. We believed that both denoising and segmentation models required a strong encoder to capture deeper ECG characteristic waveform representations. Thus, the encoder of each model was learned from the current task and inherits the knowledge of another task. As for the data corruption concern between the two tasks, when splitting the training, validation, and testing datasets for the two tasks, we ensured that the training set from one task will not be corrupted by another task’s validation and test set.

### 2.2 Data Collection and Processing

#### 2.2.1 Development Data Preparation

**FIGURE 5:**
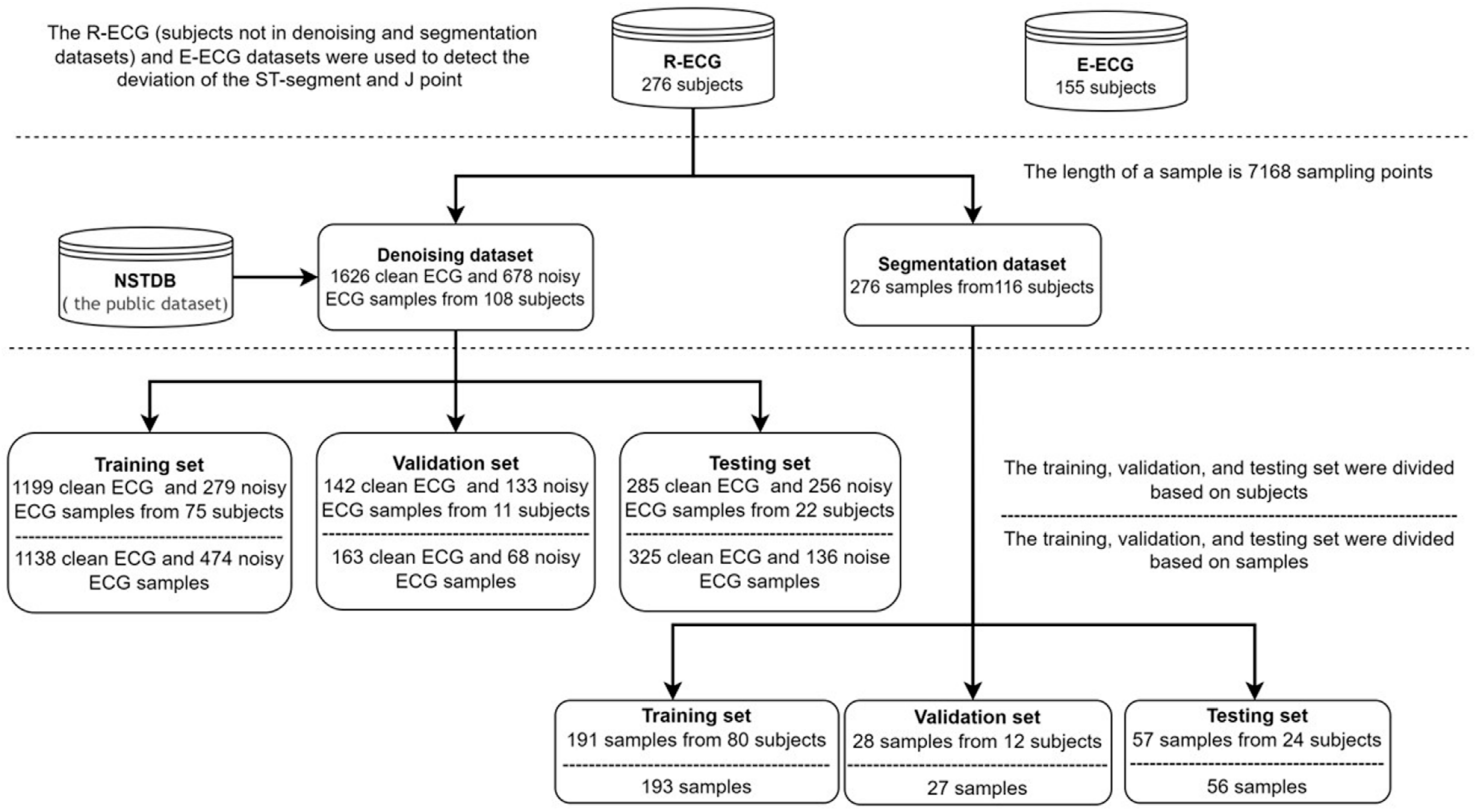
The structure of our datasets.

In this study, our ECG data comprised retrospective data from adult patients (age ≥18 years). We collected two Holter ECG (paper speed, 25 mm/s; amplification, 10 mm/mV; sampling rate, 500 Hz) datasets: the R-ECG and the E-ECG. The R-ECG dataset was used to develop the entire system, involving 276 12-lead Holter ECG records from the Department of Cardiovascular Medicine, Ruijin Hospital, Shanghai Jiao Tong University School of Medicine. The E-ECG dataset, as the external test dataset, was collected from the Department of Cardiovascular Medicine Ruijin Hospital Yuanyang Brunch, involving 155 12-lead Holter ECG records. All the Holter data were recorded using the same Holter electrocardiograph device. All subjects wore the Holter monitoring device for at least 12 h. The age distribution of the R-ECG dataset was 62.79 ± 14.78 years, with female and male percentages of 50.86 and 49.14%, respectively, in the R-ECG dataset. In the E-ECG dataset, the age distribution of 155 subjects was 63.43 ± 14.06 years, with female and male percentages of 43.87 and 56.13%, respectively (Table 1). Figure 5 provides the structure of our dataset.

**TABLE 1:** Characteristics of R-ECG and E-ECG

Characteristics	R-ECG	E-ECG
Number of subjects	276	155
Age, mean ± SD	62.79 ± 14.78	63.43 ± 14.06
Male (%)	50.86%	43.87%
Female (%)	49.14%	56.13%
Heart rate, mean ± SD	73.54 ± 11.74	74.13 ± 11.55

Anonymized data were used to ensure patient confidentiality. The algorithm team received anonymized data with only patients’ age and sex information for the subsequent model development. Informed consent was not required, because the ECG data were anonymized and deidentified.

##### 2.2.1.1 Denoising Dataset

We built a mixed noise dataset to eliminate the various noise types in the Holter ECG, which included the following:

i. The MIT-BIH Noise Stress Test Database (NSTDB) contains two noisy leads with a length of 650,000 sampling points and 360 Hz with three common nose types: muscle artifacts, electrode motion, and baseline wander (Moody et al., 1984; Goldberger et al., 2000). The data were resampled to 500 Hz to match our dataset standard.
ii. The Holter noise dataset was selected from 107 subjects, including clean and noisy signals. Each period of the signals lasted approximately 5 min.
iii. Holter noise signals were collected from daily exercise such as jogging, climbing stairs, sitting, walking etc. These data lasted approximately 2 h and were recorded from one subject. This dataset (recorded in 12 leads with 500 Hz sampling rate) represents noise types produced from daily exercise to some extent.

The generation of noisy signals is as follows:

$$Synthesized\;noise\;ECG\;=\;clean\_ECG+\;\alpha_{1}\times \;noise\_ECG_{1}+\alpha_{2}\times noise\_ECG_{2}$$

where clean_ECG and noise_ECG were cropped from clean and noise period signals separately under the same lengths; the period was randomly cropped during training and fixed cropped during validation and testing. In addition, $\alpha_{1}\;and\;\alpha_{2\;}$ were randomly generated between 0–0.5 during training, using a fixed random seed during the validation and test stages. The synthesized noise ECG was used as the input and the clean ECG was the ground truth of the model.

The de-noising dataset contains 1626 clean samples and 678 noise samples from 108 subjects, the length of a sample is 7168 sampling points (14.336 s). In the inter-analysis, the data of the 107 subjects were randomly grouped by a 7:1:2 ratio into training set (n = 75), validation set (n = 10), and testing set (n = 22). And in the intra-analysis, the data of 1626 clean samples and 678 noise samples were randomly grouped by a 7:1:2 ratio into training set (1138 clean, 474 noise), validation set (163 clean, 68 noise), and testing set (136 clean, 325 noise).

##### 2.2.1.2 QRS Complex Segmentation Dataset and Annotation Creation

The QRS complex segmentation dataset contains 276 samples from 116 subjects, with a sample length of 7168 sampling points (14.336 s). In the inter-analysis, the data of the 116 subjects were randomly grouped in a 7:1:2 ratio into the training set (*n* = 80), validation set (*n* = 12), and testing set (*n* = 24). In the intra-analysis, the data of 276 samples were randomly grouped in a 7:1:2 ratio into a training set (*n* = 193), validation set (*n* = 27), and testing set (*n* = 56).

This dataset was labeled by a primary cardiologist and a post-graduate student and then reviewed by two senior cardiologists. Two labels were created to annotate the QRS complex: Calculated-QRS (CQRS) and Noised-QRS (NQRS). CQRS denotes that the ECG signal quality of the currently labeled heartbeat is sufficient to calculate the amplitude of the ST-segment. In contrast, NQRS indicates that the current heartbeat will be culled from the calculation process because noise inference around the currently labeled heartbeat will influence the calculation of the ST-segment and J point amplitude. Further, Not-QRS (NOQRS) is used to mark points that do not belong to the QRS complex.

#### 2.2.2 Prediction Post-processing

To determine the position of the J point to confirm the location of the ST segment and isoelectric reference line (IRL), to then calculate the ST-segment and J point amplitude of deviation, we labeled QRS complexes beats by beats. The ST segment was defined from the J point to 60–80 ms after the J point. And We used the position of the Q-Q interval of each heartbeat as the IRL:

$$IRL_{\left(i,l\right)}\;=\;\frac{\left(Q_{\left(i,l\right)}+\;Q_{\left(i+1,l\right)}\right)}{2}$$

where i denotes the heart beat number, l the lead number, and Q the onset point of the QRS complex.

The position of the ST segment changes with the heart rate (HR). As the heart rate increases, the ST-segment shortens. The position of the ST-segment should be adjusted by the HR, as follows (Smrdel and Jager, 2008; Sharma et al., 2017):

$$ST_{i}\;=\left\{ \begin{array}{l} J_{\left(i\right)}+80\;ms,\;if\;HR_{\left(i\right)}<100\;bpm\\ J_{\left(i\right)}+72\;ms,if\;100\;bpm\leq HR_{\left(i\right)}<110\;bpm\\ J_{\left(i\right)}+64\;ms,if\;110\;bpm\leq HR_{\left(i\right)}<120\;bpm\\ J_{\left(i\right)}+60\;ms,if\;120\;bpm\leq HR_{\left(i\right)} \end{array}\right.$$

According to the anatomy of the heart, leads I, aVL, and -aVR are lateral limb leads; leads II, III, and aVF are inferior limb leads; leads V1 and V2 are septal leads; leads V3 and V4 are anterior leads; and leads V5 and V6 are anterolateral leads. On this basis, we divided them into six groups; lead aVR was divided into one group separately, while the other groups remained unchanged. Outliers are defined as follows (Crawford et al., 1999; Ibanez et al., 2018):

i. ST-segment elevation (STE): At least two adjacent leads with ST-segment elevation at J point ≥0.25 mV when a male is younger than 40  years old, ≥0.2 mV in males aged ≥40 years or ≥ 0.15 mV in females in leads V2–V3 and/or ≥0.1 mV in the other leads.
ii. ST-segment depression (STD): At least two contiguous leads in each group with ST-segment depression ≥0.05 mV.
iii. J point elevation: Compared with the earlier electrocardiogram, new J point elevation ≥0.1 mV in all leads (in the absence of V2 and V3 leads).

All outliers should last for a minimum period of 1 min after the first outlier appeared.

#### 2.2.3 Model Comparison and Validation on Public Databases

We further validated the performance of the proposed models. We chose DENS-ECG (Peimankar and Puthusserypady, 2021), FCN (Chiang et al., 2019), Unet_LUDB (Moskalenko et al., 2020), 1D CNN Unet and DRnet (Qiu et al., 2021) to compare the models’ performance on denoising and segmentation tasks. We further validated the performance of our proposed system on Long-term ST database (LTST DB) (Jager et al., 2003). The Long-term ST database contains 20–24-h ambulatory 2- or 3- lead ECG recordings sampled at 250 Hz from 80 subjects. Each record includes beat-by-beat QRS complex annotations and ST-segment measurements. In our study, the outliers were defined in line with guideline and the standards differed across leads. Therefore, the data without lead name were excluded. 46 2-lead and 3 3-lead ECG recordings were chosen as external validation. To match our standard and model input size, we chose the protocol C (Vmin = 100 μV and Tmin = 60 s) as annotation information and all data were resampled to 500 Hz.

#### 2.2.4 Statistical Analysis

The difference between the denoised and original groups before and after denoising was assessed using a paired *t*-test. The difference in segmentation model performance between the test dataset from R-ECG and E-ECG was assessed using an independent-samples *t*-test. The two-sided statistical significance was set at *p* < 0.05. All data were analyzed using IBM-SPSS^®^ version 26.0 (IBM Corp., Armonk, NY, United States, 2019).

### 2.3 Performance Evaluation

In denoising task, we chose the AdamW optimizer for 300 epochs under a cosine decay learning rate scheduler (Kingma and Ba, 2014). An initial learning rate of 0.0001, and batch size of 64 were used. The mean absolute error (MAE) was selected as the loss function. The evaluation metrics included the root mean square error decrease ( ${\text{RMSE}}_{\text{de}}$ ), improvement of signal-to-noise ratio ( $SNR_{imp}$ ), and percentage root mean square difference (PRD). ${\text{RMSE}}_{\text{de}}$ is calculated using ${\text{RMSE}}_{\text{in}}$ to reduce ${\text{RMSE}}_{\text{out}}$ , and a larger ${\text{RMSE}}_{\text{de}}$ indicates a better noise reduction performance. ${\text{RMSE}}_{\text{de}}$ was obtained using the following expression:

$$RMSE{\;}_{de}=\;RMSE_{in}-RMSE_{out}$$

$$RMSE_{in}\;=\;\sqrt{\frac{1}{N}\times \overset{N}{\underset{n=1}{\sum }}{\left(x_{i}\;-\;{\hat{x}}_{i}\right)}^{2}}$$

$$RMSE{\;}_{out}=\;\sqrt{\frac{1}{N}\times \overset{N}{\underset{n=1}{\sum }}{\left(x_{i}\;-\;{\tilde{x}}_{i}\right)}^{2}}$$

$SNR_{imp}$ is calculated using $SNR_{out}$ to reduce $SNR_{ouint}$ , and a large $SNR_{imp}$ indicates better noise reduction performance. $SNR_{imp}$ was obtained using the following expression:

$$SNR_{imp}=\;SNR_{out}-SNR_{in}$$

$$SNR{\;}_{in}=10\times {log}_{10}\left(\frac{\Sigma_{n=1}^{N}x_{i}^{2}}{\Sigma_{n=1}^{N}{\left(x_{i}-{\hat{x}}_{i}\right)}^{2}}\right)$$

$$SNR{\;}_{out}=10\times {log}_{10}\left(\frac{\sum_{n=1}^{N}x_{i}^{2}}{\Sigma_{n=1}^{N}{\left(x_{i}-{\tilde{x}}_{i}\right)}^{2}}\right)$$

The RPD measures the quality of recovery from the noise signal. A lower PRD value indicates better design quality. The RPD is expressed as follows:

$$\text{PRD}=\;\sqrt{\frac{\Sigma_{n=1}^{N}{\left(x_{i}-{\tilde{x}}_{i}\right)}^{2}}{\Sigma_{n=1}^{N}x_{i}^{2}}}\times 100,$$

where $x_{i}$ is the value of sampling point i in the clean signal, and ${\hat{x}}_{i}$ is the value of sampling point i in the input noise signal. ${\tilde{x}}_{i}$ is the value of sampling point i in the output denoised signal, and N is the length of the ECG signal.

In the segmentation task, the optimizer was AdamW for 300 epochs using a cosine decay learning rate scheduler. And initial learning rate of 0.0001 and batch size of 64 were used. The loss function chosen was cross-entropy loss function. This study used precision, recall, and $F_{1}$ are defined as follows:

$$Precision\;=\;\frac{True\;Positive}{True\;Postive\;+\;False\;Postive}$$

$$Recall\;=\;\frac{True\;Postive}{True\;Postive\;+\;False\;Negative}$$

$$F_{1}\;=\;2\times \frac{Precision\;\times \;Recall}{Precision\;+\;Recall}$$

where each sampling point is calculated individually.

## 3 Results

### 3.1 Denoising Model

**FIGURE 6:**
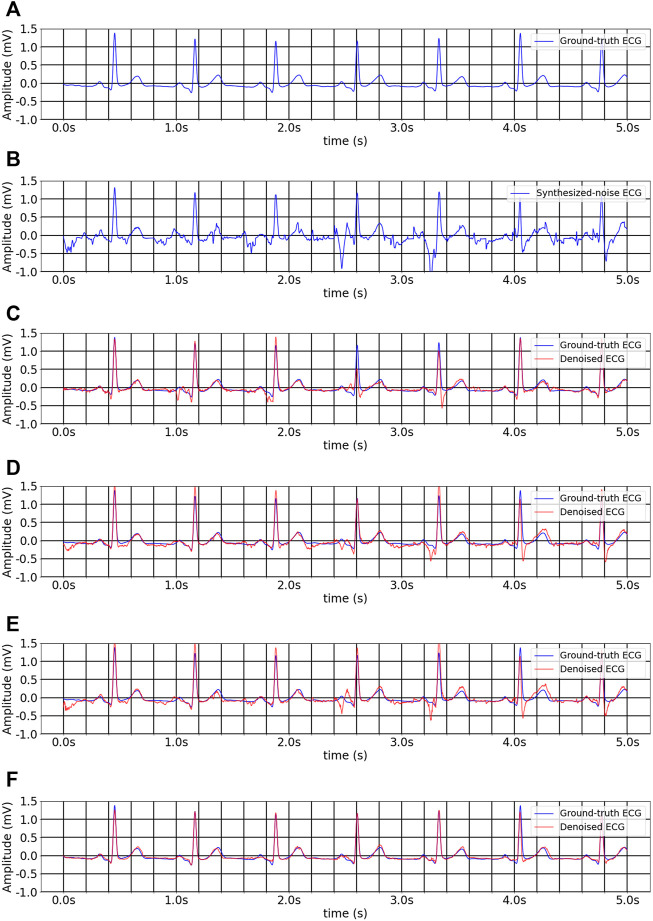
The inter-analysis denoising results of different methods on multitask inheritance training scheme. **(A)** Ground-truth ECG. **(B)** Noise-convolved ECG. **(C)** Denoised ECG by 1D CNN Unet. **(D)** Denoised ECG by FCN. **(E)** Denoised ECG by Unet_LUDB. **(F)** Denoised ECG by EBTnet.

We compared the performance between our model and the comparison models in inter- and intra-analyses, as well as in one-stage and two-stage (shown in Table 2). All four encoder and decoder models gain a better performance on multitask inheritance training scheme than training from scrath in both inter- and intra-analyses. All models gained better performance of $RMSE{\;}_{de}$ , $SNR_{imp},\;$ and PRD on intra-analysis than inter-analysis in both training schemes. In inter-analysis, compared with other models, our model achieved better performances in both one- and two-stage training schemes with $RMSE{\;}_{de}$ , $SNR_{imp},\;$ and PRD values of 0.074, 9.851, and 16.550 and 0.078, 10.903, and 14.726, respectively. Figure 6 shows the inter-analysis denoising results of different methods on multitask inheritance training scheme. The Five-fold Cross validation of inter-analysis in denoising task is shown in Supplementary Tables S3, S4.

**TABLE 2:** The comparison results of denoising models.

	Model	Training from scratch			Multitask inheritance training		
		RMSE_de_	SNR_imp_	PRD	RMSE_de_	SNR_imp_	PRD
Inter-analysis	DENS_ECG	0.028	2.546	38.541	-	-	-
	FCN	0.045	4.689	30.117	0.068**(+0.022**)	5.079**(+0.390)**	28.791**(-1.326)**
	Unet_LUDB	0.058	6.625	24.099	0.062**(+0.004**)	7.323**(+0.698)**	22.236**(-1.863)**
	1D CNN Unet	0.065	7.959	20.668	0.069**(+0.004)**	8.775**(+0.816)**	18.814**(-1.854)**
	1D CNN Unet + DRnet	0.067	0.353	19.844	-	-	-
	EBTnet	**0.071**	**9.269**	**17.774**	**0.074(+0.003)**	**10.006(+0.737)**	**16.327(-1.447)**
Intra-analysis	DENS_ECG	0.058	6.541	35.842	-	-	-
	FCN	0.068	8.409	28.908	0.070**(+0.002)**	9.049**(+0.640)**	26.852**(-2.056)**
	Unet_LUDB	0.062	7.255	22.322	0.066**(+0.004)**	8.072**(+0.817)**	20.400**(-1.922)**
	1D CNN Unet	0.068	8.790	18.363	0.073**(+0.005)**	9.672**(+0.882)**	16.967**(-1.396)**
	1D CNN Unet + DRnet	0.072	0.369	17.599	-	-	-
	EBTnet	**0.074**	**9.851**	**16.550**	**0.078(+0.004)**	**10.903(+1.052)**	**14.726(-1.824)**

Inter-analysis: The training, validation, and testing set were divided based on subjects.

Intra-analysis: The training, validation, and testing set were divided based on samples.

The bold values not in parentheses are the results of our model (EBTnet). And the bold values in parentheses indicate that the results of multi-task inheritance training are better than the results of training from scratch.

**FIGURE 7:**
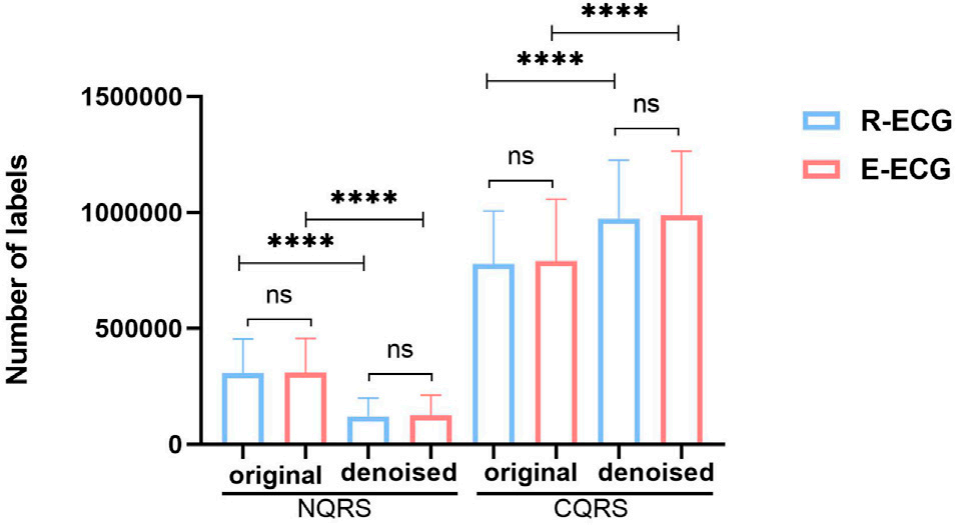
The distribution of NQRS and CQRS before and after denoising in R-ECG and E-ECG datasets. Data are expressed as mean ± SD. The difference between un-denoise and denoise groups was analyzed by paired *t*-test, and the difference between R-ECG and E-ECG was analyzed by independent-samples *t*-test. *p < 0.05, **p < 0.01, ***p < 0.001, ****p < 0.0001, and ns denoted no significance difference.

We then compared the distribution of NQRS and CQRS between original ECG signals (original group) and denoised ECG signals (denoised group) in the same dataset (Figure 7). The denoised group showed significantly more CQRS labels and less NQRS lables than the original group (*p* < 0.0001). In this work, we demonstrated that the great performance of our denoising model and the impact of signals quality on the segmentation model results. Good signal quality is essential to improve the performance of segmentation model.

### 3.2 QRS Complex Segmentation Model

**FIGURE 8:**
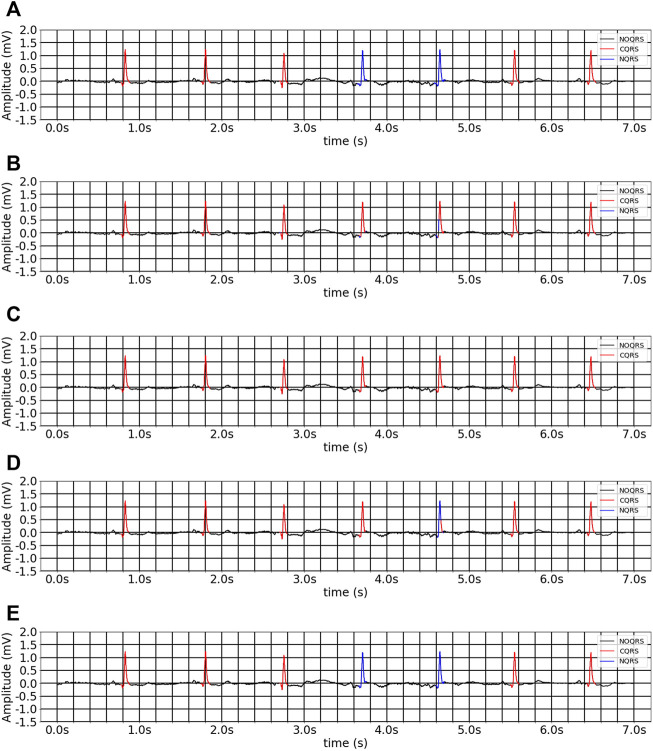
The inter-analysis segmentation results of different methods on multitask inheritance training scheme. **(A)** Ground-truth ECG. **(B)** 1D CNN Unet. **(C)** FCN. **(D)** Unet_LUDB. **(E)** EBTnet.

Tables 3, 4 present the segmentation performances between our model and the comparison models in inter- and intra-analyses, as well as training from scratch and multitask inheritance training schemes. All four encoder and decoder models performed better in the multitask inheritance training scheme than in the training from scratch in both inter- and intra-analyses. Our model achieved better performances than the other models in both inter- and intra-analyses. The precision, recall, and F1 of CQRS in the inter-analysis were 96.00, 93.06, and 93.17%, respectively. The precision, recall, and F1 of CQRS in the intra-analysis were 95.68, 96.04, and 95.86%, respectively. Figure 8 shows the inter-analysis segmentation results of different methods on multitask inheritance training scheme. The results showed that our model was sufficiently accurate in distinguishing the QRS complex, which laid the foundation for our subsequent processing. The Five-fold Cross validation of inter-analysis in segmentation task is shown in Supplementary Tables S3, S4.

**TABLE 3:** The comparison results of segmentation models in the inter-analysis.

Model	Label	Training from scratch			Multitask inheritance training		
		F1 (%)	Precision (%)	Recall (%)	F1 (%)	Precision (%)	Recall (%)
DENS_ECG	NOQRS	95.41	96.46	94.37	-	-	-
	CQRS	60.99	53.23	71.40	-	-	-
	NQRS	0.00	0.00	0.00	-	-	-
DRNET	NOQRS	99.21	99.44	98.97	-	-	-
	CQRS	89.64	87.00	92.44	-	-	-
	NQRS	42.35	45.61	39.53	-	-	-
FCN	NOQRS	99.33	99.29	99.38	99.30	98.96	99.65
	CQRS	90.04	88.53	91.61	91.55	94.76	88.55
	NQRS	42.08	51.68	35.49	45.38	43.95	46.91
Unet_LUDB	NOQRS	99.41	99.33	99.49	99.36	99.13	99.58
	CQRS	93.79	91.74	95.93	94.06	92.96	95.19
	NQRS	22.24	70.33	13.21	24.59	77.56	14.61
1D CNN Unet	NOQRS	99.50	99.56	99.44	99.51	99.45	99.56
	CQRS	93.22	93.13	93.31	94.48	95.17	93.80
	NQRS	62.45	60.36	64.70	64.16	62.33	66.11
**EBTnet**	NOQRS	99.47	99.53	99.40	99.52	99.44	99.61
	CQRS	93.83	94.50	93.17	**94.51**	**96.00**	**93.06**
	NQRS	69.62	64.07	76.24	71.85	68.50	75.54

The bold values not in parentheses are the results of our model (EBTnet). And the bold values in parentheses indicate that the results of multi-task inheritance training are better than the results of training from scratch.

**TABLE 4:** The comparison results of segmentation models in the intra-analysis.

Model	Label	Training from scratch			Multitask inheritance training		
		F1 (%)	Precision (%)	Recall (%)	F1 (%)	Precision (%)	Recall (%)
DENS_ECG	NOQRS	90.87	97.39	85.18	-	-	-
	CQRS	48.57	34.78	80.45	-	-	-
	NQRS	0.00	0.00	0.00	-	-	-
DRNET	NOQRS	99.21	98.93	99.48	-	-	-
	CQRS	89.59	91.58	87.69	-	-	-
	NQRS	45.99	47.65	44.45	-	-	-
FCN	NOQRS	99.34	99.18	99.50	99.29	99.30	99.29
	CQRS	91.52	89.83	93.29	93.56	92.07	95.11
	NQRS	45.90	68.50	34.52	49.98	69.41	39.05
Unet_LUDB	NOQRS	99.35	99.47	99.23	99.25	99.14	99.35
	CQRS	91.36	86.93	96.27	93.23	91.52	95.00
	NQRS	27.81	49.94	19.27	30.07	72.04	19.00
1D CNN Unet	NOQRS	99.50	99.54	94.63	99.54	99.58	99.51
	CQRS	94.63	94.31	94.96	95.21	94.15	96.29
	NQRS	71.23	74.43	68.31	73.59	80.37	67.87
**EBTnet**	NOQRS	99.57	99.52	99.63	99.61	99.53	99.70
	CQRS	95.38	95.43	95.34	**95.86**	**95.68**	**96.04**
	NQRS	76.76	79.75	73.99	78.75	85.70	72.84

The bold values not in parentheses are the results of our model (EBTnet). And the bold values in parentheses indicate that the results of multi-task inheritance training are better than the results of training from scratch.

### 3.3 Model’s Prediction Result

Table 5 presents the statistical outcome of our model’s prediction of STD and STE in every lead group of the-ECG and R-ECG test datasets. From the R-ECG dataset, our model detected STD in 2 patients in the lateral limb leads (I, aVL), 100 patients in the inferior limb leads (II, III, aVF), 11 patients in the aVR lead, 6 patients in the septal leads (V1, V2), 19 patients in the anterior leads (V3, V4), and 97 patients in the anterolateral leads (V5,V6). Four patients had inferior leads (II, III, aVF),3 patients had aVR lead, 3 patients had septal leads (V1, V2), 4 patients had anterior leads (V3, V4), and 1 patients had anterolateral leads (V5,V6) with STE. In the E-ECG dataset, our model detected STD in 2 patients in the lateral limb leads (I, aVL), 23 patients in the inferior limb leads (II, III, aVF),1 patients in the aVR lead, 2 patients in the septal leads (V1, V2), 4 patients in the anterior leads (V3, V4), and 20 patients in the anterolateral leads (V5,V6). One patients had inferior leads (II, III, aVF), 1 patient had septal leads (V1, V2), 2 patient had anterior leads (V3, V4) with STE.

**TABLE 5:** The distribution of the ST-segment depression and elevation in every lead group.

Datasets	Type	I, aVL	II, III, aVF	aVR	V1, V2	V3, V4	V5, V6
R-ECG	STD	2	100	11	6	19	97
	STE	0	4	3	3	4	1
E-ECG	STD	1	23	1	2	4	20
	STE	0	1	0	1	2	0

The prediction of the model was then double-checked to ensure that the outliers were correct (Table 6). In the R-ECG dataset, 103 patients with STD and 10 patients with STE were detected with positive predictive values of 80.6 and 60%, respectively. In the E-ECG dataset, 68 patients with STD and 4 patients with STE were detected with positive predictive values of 76.5 and 50%, respectively. The performance of our model on LTST DB is shown in Supplementary Table S5. And our model achieved positive predictive values (precision) of STD and STE with 97.37 and 82.35%, respectively. This result shows the robustness and generalization of our model.

**TABLE 6:** The result of cardiologist’s manual verification to validate the result of our model.

	STD		STE	
	Our system	Cardiologist	Our system	Cardiologist
R-ECG	103	83	10	6
E-ECG	68	52	4	2

## 4 Discussion

With the rapid development of computer vision and its in-depth application in the medical field, we discovered that AI can capture higher-dimensional information that is different from human thinking habits. A medical student must study for several years before becoming a physician. Qualified cardiologists require substantial professional training and experience to develop the ability to identify complicated ECG information independently. Furthermore, objective issues such as the unequal distribution of medical resources may affect the diagnosis quality. In contrast to doctors’ traditional learning methods, AI shows excellent homogeneity and accuracy, potentially narrowing the gap between outstanding physicians and rural doctors. Our previous research (Du et al., 2021) proposed an FM-ECG AI-based model to identify various cardiac abnormalities using 12-lead standard ECG data, with ECG images as the model input. It can also prove that AI can discover more information hidden in subtle ECG waveform changes, or that AI is a microscope in the world of data.

Some studies divided their datasets based on samples (Zhao et al., 2020), while others based on subjects (Xiao et al., 2018; Cho et al., 2020; Makimoto et al., 2020; Martin et al., 2021). In our study, we compared inter- and intra-analyses. Our models achieved impressive performances in both inter- and intra-analyses. The models’ performance on the intra-analysis of denoising and segmentation was better than inter-analysis. However, splitting datasets based on samples may have cross-contaminated the training, validation, and testing datasets, particularly in standard 10s 12-lead ECG. Therefore, we preferred the inter-analysis results.

Based on our research, we wanted to further explore the application value of AI algorithms in Holter ECG, thus, we developed an automatic system to detect ST-segment and J point using Holter ECG data. To learn characteristic waveform representations from ECG signals, we proposed a 1D bidirectional SWT Block that employs a window-based transformer mechanism for signal data. We discovered that using only one time-series dimension is sufficient for position embedding in a 1D bidirectional SWT Block, which preserves the properties of the ECG signal and brings it closer to the transformer’s native input. According to the results, our models outperformed the other models in both denoise and segmentation tasks. The denoising model achieved $RMSE{\;}_{de}$ , $SNR_{imp},\;$ and PRD values of 0.074, 10.006, and 16.327, respectively. Our segmentation model achieved precision, recall, and F1 scores of 94.51, 96.00, and 93.06%, respectively. These result reveals that developing a high specificity model to detect ST-segment deviation and J point elevation is possible. Hypothesizing that AI explores higher-dimension information that humans cannot paraphrase and AI can provide more novel ECG digital labels that are different from our knowledge systems to diagnose cardiac disease are reasonable.

The Holter ECG is recorded for a long time, and dividing it into a image every 10 s as model input would require a lot of computing resources. Therefore, we chose a 1D original ECG signal as the model input. Another advantage for using a 1D signal is that it contains the most primitive unprocessed information, whereas 12-lead ECG images are pre-processed by its ECG recording machine.

Prior deep learning studies have achieved strong performances in clinical medicine (Hamet and Tremblay, 2017). With the rapid development of mobile and wearable ECG technologies, several excellent ECG algorithms have emerged (Attia et al., 2019). Most existing AI-based ECG studies use public data sets to train their models. Unexpectedly, when applied in the clinical environment, the performance of the model still cannot satisfy clinical demands. To a certain extent, this can be attributed to the quality of real-world ECG data, which are more complex and variable than public datasets. Caused by daily activities such as body movement and clothing friction while wearing the ECG recorder, particularly the Holter recorder, more interfered signals would be in the 1D original ECG data. However, it requires high-quality signal data to precisely detect subtle changes in J point and ST-segment deviation. Therefore, we collected Holter ECG from the Ruijin Hospital, Shanghai Jiao Tong University School of Medicine. Then, we proposed a denoising model to reduce the disturbance of the interfered signals. We compared the distribution of NQRS and CQRS before and after denoising using the same dataset (Figure 7). A significant difference was observed between the two groups. The denoised group showed more CQRS and less NQRS labels than the original group (*p* < 0.0001). These results suggest that our denoising model has sufficient capacity to handle noisy signals and is conducive to the subsequent detection accuracy. There is no significant difference between R-ECG and E-ECG in each group, indicating that our model is sufficiently robust enough to handle different datasets.

Since AI has been applied to ECG diagnosis in recent years, arrhythmia has attracted the attention of several research teams. Andrew et al. (Hannun et al., 2019) used a deep neural network to analyze ECG data collected by a single lead ambulatory ECG monitoring device, and the performance of their model was better than that of professional physicians. Some researchers have developed a CNN deep learning algorithm to classify AF, I-AVB, left and right bundle branch blocks, atrial premature beats, and premature ventricular contraction on standard 12-lead ECG records (Oh et al., 2018; Jeong and Lim, 2021). However, in terms of shifting the detection yield to myocardial ischemia and MI, however, certain flaws have been encountered. Arrhythmia can be diagnosed with two or three leads, whereas myocardial ischemia requires at least 12 leads to affirm that the myocardial damaged position, as ECG waveforms can be different in each lead when coronary artery damage occurs in different locations. Moreover, the dynamic change of the ST-segment in myocardial ischemia and MI is difficult to be captured by standard 12 leads ECG continuously, particularly in unstable angina.

To precisely identify the IHD, the proposed model is designed to recognize the QRS complex to calculate the ST-segment and J point deviation on 12 leads Holter ECG. Table 5 presents the statistical results of our model. The J point masks the end of the ventricular depolarization and the start of repolarization. The deviation of the J point generally does not exceed 0.1 mV, it might suggest cardiac injury otherwise. The precise positioning of the J point is also of great significance. For example, it can be used to calculate PJ interval, which indicates the conduction abnormalities when it is prolonged more than 0.27 s. Although we did not find patients with J point elevation in our dataset, we found patients with STE and STD, which proves that our system can positioning J point with excellent ability. Inferior wall myocardial injuries are more common in patients with myocardial injuries (Warner and Tivakaran, 2021). More STDs were detected in the inferior leads (II, III, aVF) (Shah et al., 1983). Although inferior myocardial infarction has a better prognosis than other cardiac locations, we should note that it can be associated with right ventricular infarction, which portends a worse outcome. STE was always detected during the super-acute and acute periods of STEMI; therefore, we captured less STE in our dataset than STD. We double-checked the model’s prediction to confirm whether the outliers were correct (Table 6). In the R-ECG dataset, STD and STE were detected with positive predictive values of 76.9 and 64%, respectively. In the E-ECG dataset, STD and STE were detected with positive predictive values of 85.7 and 55.5%, respectively. STEMI accounts for 30% of acute coronary syndromes, whereas acute coronary syndrome without significant STE accounts for 70%. Patients with STD accounted for approximately 31% of acute coronary syndromes without significant STE, whereas STD combined with T-wave inversions accounted for 16% (Bhatt et al., 2022). Our results are consistent with the distribution of disease characteristics. STE can present as MI, acute pericarditis, myocarditis, vasculitis, and hyperkalemia. However, the cases presenting with STE were assumed to be STEMI. STEMI is the primary cause of STE and is a medical emergency that requires prompt recognition and treatment (Chandra et al., 2011). Therefore, fewer STEs were found among Holter-wearing patients. In our dataset, the number of patients with STE was small, and the results may have improved if the dataset had a larger positive sample size.

For unstable angina and stable angina pectoris, approximately half of the 12-lead standard ECG is normal when the diseases is resting. Holter can record ECG for at least 24 h, and the ischemic changes shown on ECG at a corresponding time during chest pain attacks can determine the diagnosis of angina. In addition, painless myocardial ischemia can be detected using a Holter ECG recorder. Moreover, it would benefit patients with slight myocardial ischemic symptoms who have a high risk of cardiovascular or sudden cardiac death. Although Holter has the above advantages for detecting myocardial ischemia, it is rarely applied to automated myocardial ischemia monitoring. Owing to the existing Holter equipment failure to detect ST-segment with high precision, the result of the deviation of the ST-segment does not help in diagnosis. That is, diagnosing silent myocardial ischemia is still challenging since physicians cannot analyze each heartbeat from 24 h of Holter ECG data.

The proposed system can provide more accurate information with an excellent ability to handle large amounts of data to cardiovascular system regarding whether the patients suffer from myocardial ischemia while wearing Holter ECG recorders. Furthermore, the accurate detection of the ST-segment and J point may be a powerful force in resolving the excessive false alarms that afflict current ST monitoring software.

### 4.1 Limitation

Several limitations of this study should be noted. First, it was performed at a single center in Shanghai, China. Using external real-word data sets from other regions is necessary for further verification and analysis to ensure the validity of our AI model worldwide. Second, the proposed model trained with ECG data only incorporated age, sex, with biomarkers, medicines, or other history information. Additional patient data may have further improved the diagnostic value of our model and led to the discovery of previously unknown conscious ECG information. Third, rather than using the gold standard of coronary heart disease, such as coronary angiography, our system’s conclusions were confirmed only by cardiologists. In terms of models, the proposed denoising model performes well in some inferred signals, but it is powerless with severe noise signals, such as part of the lead falling off or vigorous clothing friction. Moreover, to a certain degree, our model’s diagnostic result may lack continuity and the period of STD is discontinuous. This is because of our model judgment rules: an abnormal condition is assessed as the associated abnormal label and noted on the table only if it lasts for at least 1 min. The present QRS complex is not be included in the computation if the model deems a QRS complex as NQRS. Therefore, once an NQRS label appears in a segment of the ST-segment abnormal ECG signal, our results show the characteristics of the discontinuous distribution.

### 4.2 Future Study

We have investigated the possibility of applying AI to analyze ECG images and 1D signals. Future directions are related to improving the establishment of the Holter ECG dataset and merging of illness information in more dimensions. First, more information about the patient history and various inspection results will be recorded. Patients who have a gold standard for CHD will be chosen as the control group to verify our results. Other information such as echocardiogram, electrolyte, blood lipid level, blood pressure, and blood sugar can provide model more dimensional information to diagnose and further predict potential diseases. Second, in the current study, we failed to find patients with J point elevation, but we expect that with additional Holter ECG data, we can screen patients with J point elevation and follow them for years. We may then look for a link between J point elevation and heart diseases end events, as well as predict critical events such as ventricular fibrillation and SCD. Third, in terms of AI models, we will build a multi-label AI model to classify arrhythmia, MI, and other disorders such as myocarditis and hyperkalemia using long-term ECG data. Finally, future studies, particularly large multicenter prospective cohort studies, would be conducted to assess the prediction level of the AI model.

## 5 Conclusion

In conclusion, we proposed a transformer-structure-based automatic system combining denoising and segmentation modules, which can be applied to identify ST-segment and J point abnormalities in patients from long-term Holter ECG data. The proposed system has the potential to assist in clinical decisions while reducing the burden on doctors with fewer medical resources.

## Data Availability

The raw data supporting the conclusions of this article will be made available by the authors. And the original code was released in GitHub at https://github.com/caoqing-ruijing/ST_Holter.
