# Supplementary material for: Using Multi-Task Learning-Based Framework to Detect ST-Segment and J-Point Deviation From Holter
Source: Front Physiol. 2022 Jun 29;13:912739. doi: 10.3389/fphys.2022.912739 (PMC9277481; doi:10.3389/fphys.2022.912739)
Supplement: Supplementary file 1 [file Table1.DOCX]

**Supplementary Table1.** Ablations results of inter-analysis at both denoising task and segmentation task using the EBTnet with different window size and depths at each stage. All the experiments are training from scratch under the same hyper-parameter setting. In both tasks, the 112 setting outperforms the other settings in terms of window size. At depths at three stages setting, both the encoder and the decoder use the same depths. The results in both tasks do not improve significantly as the depths increase in the second and third stages. As a result, we keep the 2,2,2 setting.

| Window size | Depths at 3 stages | Denoising Task | | |  | Segmentation Task | | |
| --- | --- | --- | --- | --- | --- | --- | --- | --- |
|  |  | RMSE_de_ | SNR_imp_ | PRD |  | F1 of NOQRS（%） | F1 of CQRS（%） | F1 of NQRS（%） |
| 54 | 2, 2, 2 | 0.0701 | 9.0266 | 18.2766 |  | 99.507 | 93.354 | 68.192 |
|  | 2, 3, 2 | 0.0702 | 9.0419 | 18.1661 |  | 99.381 | 93.471 | 68.381 |
|  | 2, 3, 3 | 0.0703 | 9.0741 | 18.0991 |  | 99.395 | 93.658 | 68.468 |
| 112 | 2, 2, 2 | 0.0710 | 9.2690 | 17.7740 |  | 99.472 | 93.833 | 69.622 |
|  | 2, 3, 2 | 0.0710 | 9.2346 | 17.7676 |  | 99.392 | 93.542 | 68.858 |
|  | 2, 3, 3 | 0.0710 | 9.2296 | 17.7778 |  | 99.373 | 93.504 | 69.736 |
| 224 | 2, 2, 2 | 0.0707 | 9.1603 | 17.9975 |  | 99.506 | 93.354 | 69.083 |
|  | 2, 3, 2 | 0.0707 | 9.1573 | 17.9264 |  | 99.505 | 93.581 | 69.239 |
|  | 2, 3, 3 | 0.0709 | 9.2078 | 17.8994 |  | 99.506 | 93.387 | 69.374 |
| 448 | 2, 2, 2 | 0.0701 | 9.0361 | 18.2567 |  | 99.482 | 93.358 | 68.919 |
|  | 2, 3, 2 | 0.0701 | 9.0156 | 18.2999 |  | 99.411 | 93.658 | 69.086 |
|  | 2, 3, 3 | 0.0703 | 9.0665 | 18.1929 |  | 99.386 | 93.592 | 69.189 |

**Supplementary Table2.** Ablations results of inter-analysis at both denoising task and segmentation task using the EBTnet with different block type and window size. The experiments follow the same environment setting with supplementary Table1. In terms of block type, SWT stands for regular combinations, which refers to two consecutive SWT blocks with unshifted, and forwards shifted configurations, respectively. Our proposed method is Bi-SWT, which consists of three SWT blocks with unshifted, forwards shifted, and backwards shifted configurations. To compare the regular SWT with the same number of blocks. We make a SWT$\times$2 by adding another forward shifted configuration to SWT, which includes unshifted, forwards shifted, and forwards shifted configurations. To fairly comparison with regular SWT under same numbers of blocks. The results show that our Bi-SWT outperforms the competition in both tasks.

| Block type | Window size | Denoising Task | | |  | Segmentation Task | | |
| --- | --- | --- | --- | --- | --- | --- | --- | --- |
|  |  | RMSE_de_ | SNR_imp_ | PRD |  | F1 of NOQRS（%） | F1 of CQRS（%） | F1 of NQRS（%） |
| SWT | 54 | 0.0678 | 8.5111 | 19.3942 |  | 99.467 | 93.477 | 64.690 |
|  | 112 | 0.0688 | 8.7361 | 18.8982 |  | 99.504 | 93.421 | 66.175 |
|  | 224 | 0.0692 | 8.8254 | 18.7051 |  | 99.486 | 93.387 | 66.851 |
|  | 448 | 0.0686 | 8.6877 | 18.9223 |  | 99.399 | 93.497 | 65.487 |
| SWT$\times$2 | 54 | 0.0680 | 8.5568 | 19.2924 |  | 99.485 | 93.467 | 64.774 |
|  | 112 | 0.0692 | 8.8331 | 18.6884 |  | 99.482 | 93.421 | 66.851 |
|  | 224 | 0.0692 | 8.8274 | 18.7008 |  | 99.504 | 93.387 | 66.879 |
|  | 448 | 0.0690 | 8.7914 | 18.7783 |  | 99.498 | 93.231 | 65.643 |
| Bi-SWT | 54 | 0.0701 | 9.0266 | 18.2766 |  | 99.507 | 93.354 | 68.192 |
|  | 112 | 0.0710 | 9.2690 | 17.7740 |  | 99.472 | 93.833 | 69.622 |
|  | 224 | 0.0707 | 9.1603 | 17.9975 |  | 99.506 | 93.354 | 69.083 |
|  | 448 | 0.0701 | 9.0361 | 18.2567 |  | 99.482 | 93.358 | 68.919 |

**Supplementary Table 3.** The training, validation and testing sets were divided based on subjects with 7:1:2 ratio. The fold 1 was chosen to apply in other experiments.

| Folds | Denoising task | | |  | Segmentation Task | | |
| --- | --- | --- | --- | --- | --- | --- | --- |
|  | Training | Validation | Testing |  | Training | Validation | Testing |
| Fold 1 | 75 | 10 | 22 |  | 80 | 12 | 24 |
| Fold 2 | 75 | 10 | 22 |  | 80 | 13 | 23 |
| Fold 3 | 76 | 10 | 21 |  | 80 | 13 | 23 |
| Fold 4 | 75 | 11 | 21 |  | 80 | 13 | 23 |
| Fold 5 | 76 | 10 | 21 |  | 80 | 13 | 23 |

**Supplementary Table 4.** The model’s performance of five-fold in both tasks.

| Folds | Denoising task | | |  | Segmentation Task | | |
| --- | --- | --- | --- | --- | --- | --- | --- |
|  | RMSE_de_ | SNR_imp_ | PRD |  | F1 of NOQRS（%） | F1 of CQRS（%） | F1 of NQRS（%） |
| Fold 1 | 0.0712 | 9.2688 | 17.7741 |  | 99.465 | 93.826 | 69.623 |
| Fold 2 | 0.0716 | 9.3747 | 17.4834 |  | 99.439 | 94.228 | 71.258 |
| Fold 3 | 0.0707 | 9.1573 | 17.9264 |  | 99.449 | 93.848 | 67.132 |
| Fold 4 | 0.0709 | 9.2051 | 17.8281 |  | 99.452 | 94.032 | 66.936 |
| Fold 5 | 0.0719 | 9.4480 | 17.3365 |  | 99.459 | 93.805 | 72.553 |

**Supplementary Table 5.** The performance of our model on LTST DB.

|  | Ground truth | Model pred | Accuracy (%) | Precision (%) | Recall (%) | F1 (%) |
| --- | --- | --- | --- | --- | --- | --- |
| STD | 43 | 38 | 85.71 | 97.37 | 86.05 | 91.36 |
| STE | 15 | 17 | 91.84 | 82.35 | 93.33 | 87.5 |
